# Chitinolytic enzymes contribute to the pathogenicity of *Aliivibrio salmonicida* LFI1238 in the invasive phase of cold-water vibriosis

**DOI:** 10.1186/s12866-022-02590-2

**Published:** 2022-08-08

**Authors:** Anna Skåne, Per Kristian Edvardsen, Gabriele Cordara, Jennifer Sarah Maria Loose, Kira Daryl Leitl, Ute Krengel, Henning Sørum, Fatemeh Askarian, Gustav Vaaje-Kolstad

**Affiliations:** 1 Faculty of Chemistry, Biotechnology and Food Science, Norwegian University of Life Sciences (NMBU), Ås, Norway; 2 Department of Chemistry, University of Oslo, Blindern, P.O. Box 1033, NO-0315 Oslo, Norway; 3 Department of Paraclinical Sciences, Faculty of Veterinary Medicine, Norwegian University of Life Sciences (NMBU), Oslo, Norway; 4 Division of Host-Microbe Systems & Therapeutics, Department of Pediatrics, School of Medicine, UC San Diego, La Jolla, San Diego, CA USA

**Keywords:** lytic polysaccharide monooxygenase, LPMO, virulence, aliivibrio salmonicida, chitin, cold water vibriosis, vibriosis

## Abstract

**Background:**

*Aliivibrio salmonicida* is the causative agent of cold-water vibriosis in salmonids (*Oncorhynchus mykiss* and *Salmo salar* L.) and gadidae (*Gadus morhua* L.). Virulence-associated factors that are essential for the full spectrum of *A. salmonicida* pathogenicity are largely unknown. Chitin-active lytic polysaccharide monooxygenases (LPMOs) have been indicated to play roles in both chitin degradation and virulence in a variety of pathogenic bacteria but are largely unexplored in this context.

**Results:**

In the present study we investigated the role of LPMOs in the pathogenicity of *A. salmonicida* LFI238 in Atlantic salmon (*Salmo salar* L.). In vivo challenge experiments using isogenic deletion mutants of the two LPMOs encoding genes AsLPMO10A and AsLPMO10B, showed that both LPMOs, and in particular AsLPMO10B, were important in the invasive phase of cold-water vibriosis. Crystallographic analysis of the AsLPMO10B AA10 LPMO domain (to 1.4 Å resolution) revealed high structural similarity to viral fusolin, an LPMO known to enhance the virulence of insecticidal agents. Finally, exposure to Atlantic salmon serum resulted in substantial proteome re-organization of the *A. salmonicida* LPMO deletion variants compared to the wild type strain, indicating the struggle of the bacterium to adapt to the host immune components in the absence of the LPMOs.

**Conclusion:**

The present study consolidates the role of LPMOs in virulence and demonstrates that such enzymes may have more than one function.

**Supplementary Information:**

The online version contains supplementary material available at 10.1186/s12866-022-02590-2.

## Background

*Aliivibrio salmonicida* (*Vibrio salmonicida* before transfer to genus *Aliivibrio*) is the causative agent of cold-water vibriosis (CWV) in salmonids (*Oncorhynchus mykiss* and *Salmo salar* L.) and gadidae (*Gadus morhua* L.), an acute infectious disease consistent with severe hemorrhagic septicemia [1–4]. Once the pathogen enters the bloodstream [5], *A. salmonicida* can disseminate in many sites, *e.g.* sinusoids of the head kidney/lymphoid organ, leukocytes, and endothelial cells [6], and even actively proliferate in blood upon passing a latent stage [5, 7, 8]. Notably, histopathological changes caused by the bacterium are found to be associated with the bacterial burden [6]. Although CWV is under control by vaccination, virulence-associated factors that are essential for the full spectrum of *A. salmonicida* pathogenicity are largely unknown. So far, in vitro and in vivo studies have demonstrated that the salinity-sensitive quorum-sensing regulator LitR [9], LPS O-antigen [10], motility/flagellation [11], and the *lux* operon [12] are required for full virulence of *A. salmonicida*.

Chitinolytic enzymes include chitinases (glycoside hydrolases 18 and 19 (GH18 and GH19)) and lytic polysaccharide monooxygenases (LPMOs), with the latter classified in the auxiliary activities 10 family (AA10) according to classification by the Carbohydrate Active Enzymes database (CAZy [13]). Such enzymes are associated with the modification, binding, depolymerization, and catabolism of chitin [14–18]. LPMOs were discovered in 2010 [18], and thus represent a recent addition to the chitin degradation machinery. These copper-dependent, redox enzymes cleave chitin chains by an oxidative reaction and synergize with chitinases in chitin degradation reactions [18–21]. Intriguingly, genes encoding LPMOs are found in an array of pathogenic bacteria [22], and there is an extensive amount of literature implicating their role in numerous biological processes including bacterial pathogenicity [22–30]. Direct evidence for a role of LPMOs in virulence was recently published by Askarian et al., who showed that the LPMO of the opportunistic human pathogen *Pseudomonas aeruginosa*, called CbpD, was important for establishing systemic- and lung infections, where the role of the enzyme was attributed to attenuation of the terminal cascade of the complement system [31]. The latter study showed that deletion of the *cbpd* gene prevented *P. aeruginosa* from establishing a lethal systemic infection in mice and that this correlated with increased clearance of the bacterium in vivo and re-organization of the bacterial proteome in vitro. Further, it was found that an intact active site was essential for CbpD function. A somewhat different role has been proposed for the *Vibrio cholerae* LPMO, GbpA, which binds chitin and mucins, mediating bacterial colonization of epithelial cell surfaces [32]. Similar to LPMOs, chitinases have also been indicated as virulence factors. For example, *Listeria monocytogenes* ChiA was found to promote bacterial viability within the liver and spleen of mice [25], and the chitinase (ChiA) of *Legionella pneumophila* has been shown to enhance bacterial persistence in the lungs of mice in vivo [33]. Recently, it has been shown that *L. pneumophila* ChiA is involved in hydrolysis of the peptide bonds of mucin-like proteins [34], suggesting novel mechanisms of mucin degradation.

The *A. salmonicida* LFI1238 genome harbors genes encoding two family AA10 LPMOs (*AsLPMO10A, AsLPMO10B*) and one chitinase GH18 (*AsChi18A*). The two LPMOs are relatively dissimilar, showing only 20% sequence identity when aligning the catalytic domains. All three enzymes can depolymerize chitin and are important for the ability of the bacterium to utilize chitin as a nutrient source [35]. However, the authors noticed several features that could indicate additional roles of the enzymes, for instance a remarkably low chitinolytic activity of the chitinase, and the chitin-independent expression of *As*LPMO10A (this protein is one of the most abundant proteins produced by the bacterium) [35]. In addition, the whole genome sequencing analysis of *A. salmonicida* LFI1238 had previously shown several points of mutation or insertion of mobile genetic elements within crucial genes associated with the chitinolytic machinery (*e.g.* several chitinases, a chitoporin and a protein important for regulating expression of the chitin degradative loci [36]). Cumulatively, these results suggest the contribution of the chitinolytic enzymes to other or additional functions beyond chitin degradation and utilization by *A. salmonicida*. Thus, the current work set out to elucidate the putative immune evasive properties of *As*LPMO10A (A) and *As*LPMO10B (B) in *A. salmonicida* pathogenesis during CWV in Atlantic salmon. Using a series of isogenic mutants (△A, △B and △AB), we found that the LPMOs contributed to the pathogenicity of *A. salmonicida* in the invasive phase of CWV.

## Results

### Phylogenetic analysis

**Fig. 1:**
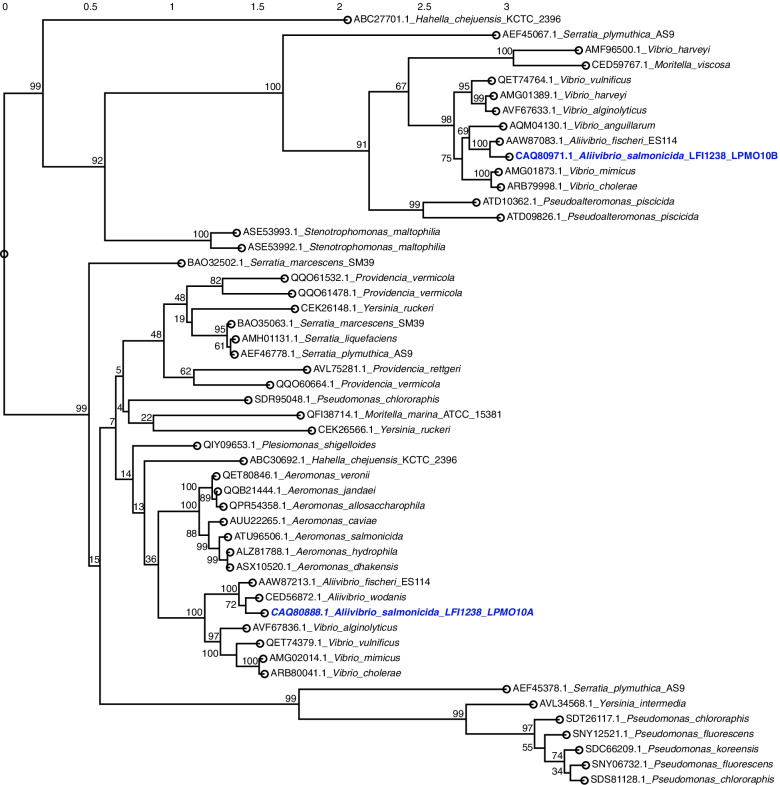
Phylogenetic tree of family AA10 LPMOs from fish pathogens. Alignment and phylogenetic reconstructions were performed using the “build” function of ETE3 v3.1.1 21 [38] that utilizes PhyML v20160115 [39]. Branch support values were computed from 100 bootstrapped trees. Refseq identifiers for the proteins are indicated next to the name of the bacterial species. The *A. salmonicida* LPMOs are indicated in blue colored bold formatting

The sequence and biochemical properties of *As*LMO10A and *As*LPMO10B have previously been biochemically characterized [35] but their putative orthologs in other fish pathogens are not known. To determine the latter and to simultaneously obtain an overview of LPMOs in bacteria associated with fish disease, the genomes of fish pathogens [37] were scanned for LPMO-encoding genes that subsequently were subjected to phylogenetic analysis (Fig. 1). The analysis showed that LPMOs are present in the majority of aerobic Gram-negative bacteria investigated, but to a lesser extent in Gram-positives. *As*LPMO10A clusters with LPMOs from a variety of bacterial families, whereas *As*LPMO10B clusters with representatives mostly restricted to the Vibrionaceae. The analysis does not show clustering indicative of horizontal gene transfer but rather indicates that the LPMO paralogs were present in an ancestral Vibrionaceae bacterium.

### Proteomic profiling

Gene deletions may induce alterations in protein regulation by the bacterium to adapt to this impairment. Such re-organization can be readily visualized by comparing the proteomic response of wild-type (WT) and gene-deletion variants confronted with host factors. Thus, comparative label-free quantitative proteomics was used to determine the putative proteomic response of wild type, ΔA, ΔB and ΔAB strains when exposed to Atlantic salmon serum (SS). The bacteria were grown to early exponential phase and incubated for 1 h in the absence or presence of SS, prior to being harvested. In total, 1725 proteins were identified, corresponding to almost half of the predicted proteome of *A. salmonicida* (Dataset 1).

**Fig. 2:**
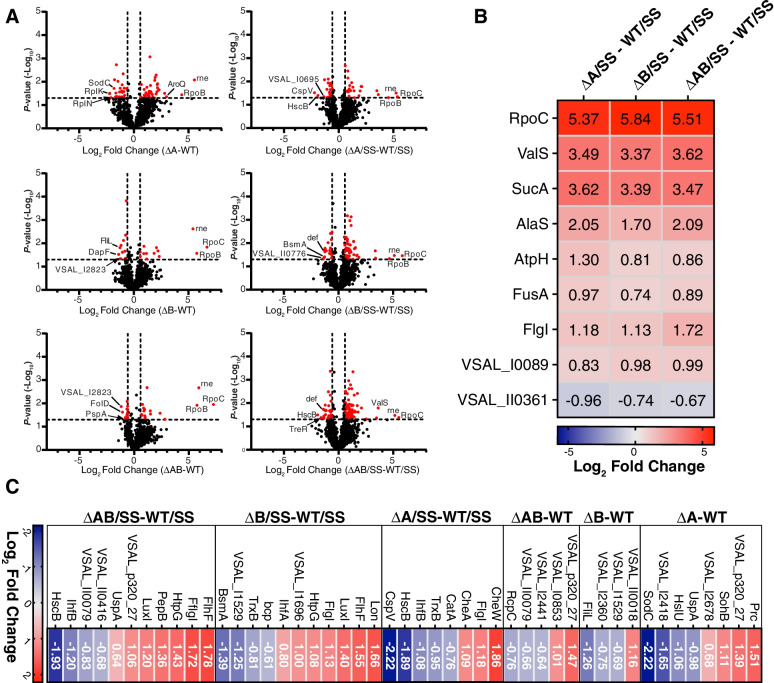
Significantly regulated proteins of *A. salmonicida* deletion variants exposed to Atlantic salmon serum. (**A**) Volcano plots showing the *p*-values of significance and log_2_ fold change values comparing ΔA, ΔB or ΔAB against WT in the absence (left panel) and presence (right panel) of salmon serum (SS). Dotted line(s) traversing the y- and x-axis indicate the significance cutoff at *p* = 0.05 (-log_10_ = 1.3) and ( ±) 1.5-fold change (log_2_ = 0.58) in protein abundance. Significance was determined by a paired two-tailed t-test. (**B**) Heatmaps showing the fold change values (log_2_) of significantly regulated proteins common for all deletion mutants. (**C**) Heatmap showing fold-change values (log_2_) of significantly regulated proteins related to motility, chemotaxis, quorum sensing, protease activity and general stress response

The whole-cell proteomes of the deletion mutants were compared to that of the wild type in the absence and presence of SS. The comparison showed significant regulation of 61 (∆A), 27 (∆B) and 32 (∆AB) and 46 (∆A) and 70 (∆B) and 94 (∆AB) in the absence or presence of SS, respectively (Fig. 2A). In the absence of SS, the most significantly upregulated protein was RpoC (DNA-directed RNA polymerase subunit) for ΔB and ΔAB and Rne (ribonuclease) for ∆A (Dataset 2). Beside RpoC, RpoB (DNA-directed RNA polymerase subunit) and Rne was found to be among the top three upregulated proteins in most of the deletion mutants (Dataset 2 and 3).

In the presence of SS, RpoC was one of the most upregulated proteins for all strains (similar to what was observed in bacteriologic medium), in addition to ValS (Valine-tRNA ligase), SucA (oxoglutarate dehydrogenase) and AlaS (Alanine-tRNA ligase; Fig. 2B). Also, several proteins related to motility, chemotaxis, quorum sensing and stress response were identified as significantly regulated (Fig. 2C, Dataset 2). The ΔA deletion strain resulted in up-regulation of CheW (chemotaxis protein), CheA (phosphorelay protein LuxU) and FlgL (flagellar P-ring protein). The latter protein was identified as up-regulated in all deletion variants after exposure to SS (Fig. 2B), whereas in absence of SS, it was downregulated in the ΔB strain (Fig. 2C). Moreover, exposure to SS resulted in up-regulation of FlhF (flagellar biosynthesis protein), LuxI (autoinducer synthesis protein) and chaperone protein HtpG in the ∆B and ∆AB strains. Proteins related to stress response were down-regulated in ΔA (*e.g.* CatA (catalase), TrxB (thioredoxin reductase), CspV (cold shock protein)) and ΔB (*e.g.* Bcp (putative peroxiredoxin), TrxB and VSAL_I1529 (putative glutaredoxin)) in presence of SS (Fig. 2C, Dataset 2). Notably, proteins with peptidase- and protease-related activity were identified as differentially regulated both in absence and presence of serum. Specifically, in absence of SS, deletion of *As*LPMO10A resulted in up-regulation of Prc (tail-specific protease) and SohB (probable protease), and down-regulation of HslU (ATP-dependent protease ATPase subunit) compared to the wild type (Fig. 2C, Dataset 2). It should be noted that HslU has an indirect protease activity as it is a subunit of the heat-shock locus HslV-HslU complex associated with the proteasome of many bacteria [40, 41].

After incubation with SS, Lon protease and PepB (peptidase B) were up-regulated in ΔA and ΔAB, respectively. The protein called BsmA, involved in cell aggregation for biofilm development, was found to be down-regulated in the ∆B deletion mutant (Fig. 2C, Dataset 2). Host integration factor subunit B (IhfB) was down-regulated in ΔA and ΔAB in presence of SS compared to the wild-type, while subunit A (IhfA) was up-regulated in ∆B compared to the wild-type (Fig. 2C, Dataset 2). Notably, the host integration factor is implicated in regulation of virulence-related factors in *V. cholerae* [42], *Vibrio vulnificus* [43] *Vibrio harveyi* [44] and *Vibrio fluvialis* [45]. Interestingly, the transposon VSAL_I0029 was up-regulated in both ∆B and ∆AB in the presence of SS. The function of this transposon is not known; however, it is located closely to a reported T6SS effector VSAL_I0031 [46]. This gene encodes a so-called MIX (Marker for type sIX) effector, and these effectors have C-terminal domains predicted to contain different antibacterial or anti-eukaryotic properties [46]. Finally, *As*LPMO10B was not detected in any samples, whereas *As*LPMO10A was observed in both the wild-type and ∆B (but not significantly regulated in any condition).

Together, these data indicate that deletion of the LPMO encoding genes in *A. salmonicida* results in a substantially altered proteome response compared to wild type. Moreover, the number of differentially regulated proteins in the ΔB and ΔAB strains were remarkably increased in the presence of SS.

### In vivo immersion challenge experiments to establish bacteremia

**Fig. 3:**
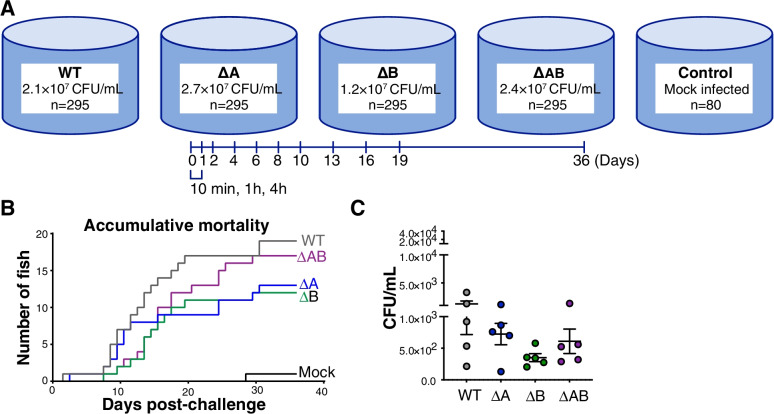
Immersion challenge to establish bacteremia. (**A**) Challenge groups and sub-lethal infection doses represented by colony forming units (CFU) pr mL *A. salmonicida* containing seawater. (**B**) Accumulative mortality. (**C**) Presence of *A. salmonicida* WT and deletion variants in blood after 10 min of exposure. Bacteria (WT, ΔA, ΔB or ΔAB) were isolated from the drawn blood of Atlantic salmon following immersion challenge. Data are shown as individual values (*n* = 5) with mean ± SEM representing colony forming units (CFU) pr mL blood

**Fig. 4:**
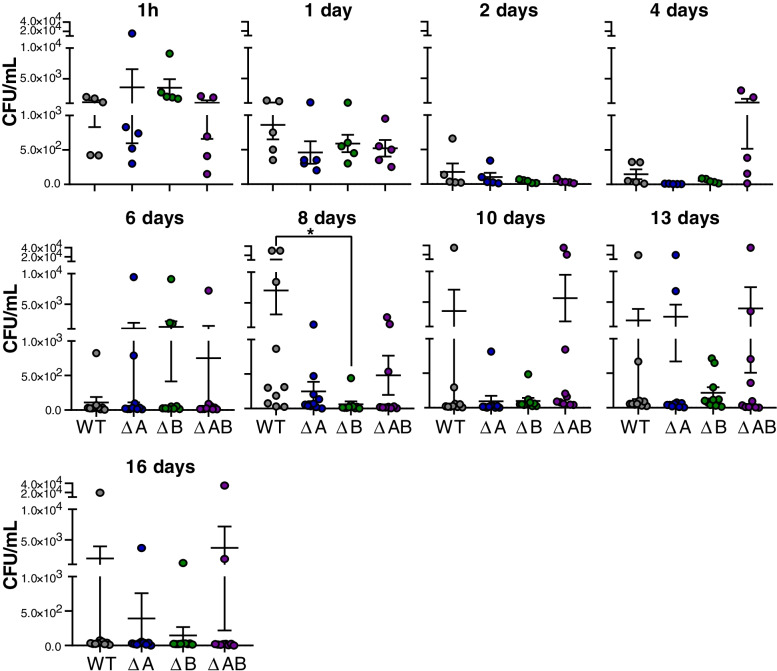
Presence of *A. salmonicida* variants in blood from 1 h to 16 days post-challenge. Bacteria (WT, ΔA, ΔB or ΔAB) were isolated from drawn whole blood of Atlantic salmon following immersion challenge. Data are shown as individual values (*n* = 5–10) with mean ± SEM representing colony forming units (CFU) pr mL blood

To provide insight into the contribution of LPMOs in the virulence properties of *A. salmonicida,* an immersion challenge was carried out using the wild type and deletion variants (Δ*A*, Δ*B,* ΔAB). In an experiment using a total of 1340 Atlantic salmon smolts, fish were immersed in a high concentration of *A. salmonicida* variants for 30 min, followed by water exchange (Fig. 3A). Immersion in approximately 1.2–2.7 × 10^7^ CFU/mL wild type and gene deletion strains resulted in a persistent bacteremia (Figs. 3 and 4) without exhaustive killing (Fig. 3B). The examined conditions resulted in a low number of accumulated mortalities (below 10%) in the wild type and deletion strains over the course of the challenge (Fig. 3B). Furthermore, the employed concentrations resulted in successful establishment of bacteremia as all sampled fish were positive for presence of *A. salmonicida* in blood 10 min post-infection (Fig. 3C). The presence of fin rot was observed evenly within all treatments but did not contribute to an extensive rate of mortality as reflected in the mock treatment (Fig. 3B).

### Bacterial burden in blood

**Fig. 5:**
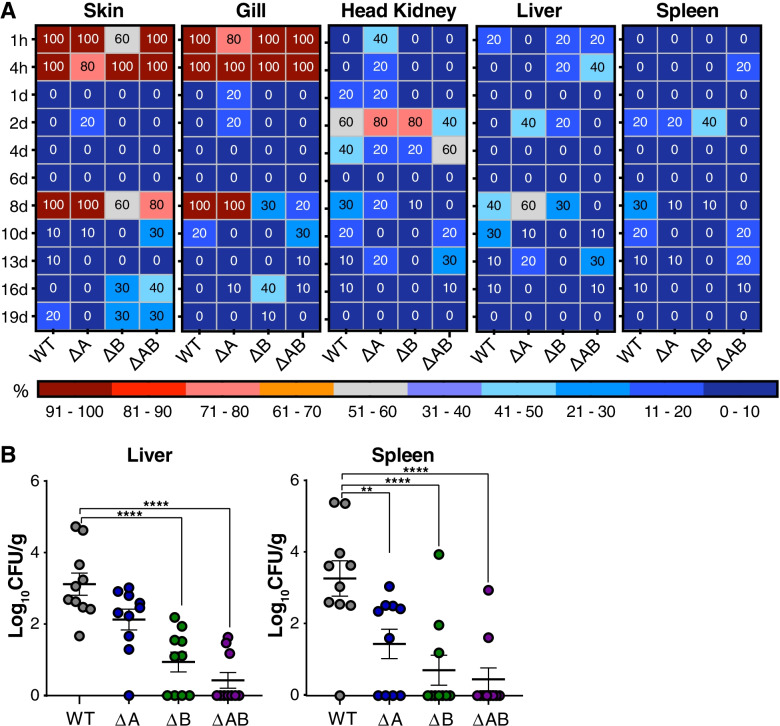
Presence of *A. salmonicida* variants from tissue and organs over the course of CWV infection. (**A**) Percentage of sampled fish that were positive for presence of *A. salmonicda* from 1 h to 19 days post immersion challenge with WT, ΔA, ΔB and ΔAB. (**B**) Estimation of bacterial loads in organs. Bacteria (WT, ΔA, ΔB or ΔAB) were isolated from the homogenized spleen or liver of Atlantic salmon following 8 days post-immersion challenge. Data are shown as individual values (*n* = 10) with mean ± SEM representing colony forming units (CFU) per gram organ

Fish challenged with wild type, ΔA, ΔB and ΔAB, and sampled at multiple time points post-challenge showed the presence of *A. salmonicida* in a various degree throughout the complete sampling period, indicating the successful establishment of CWV in our experimental condition (Figs. 4 and 5). A decrease of the bacterial number in whole salmon blood was observed between days 1–6 compared to 1 h post-challenge in wild type, ΔA, ΔB and ΔAB infected fish (Fig. 4). At 8 days post infection, the group challenged with the wild type strain showed large individual variation and a significant increase in bacterial burden compared to the ΔB mutants but not ΔA and ΔAB infected fish (Fig. 4). The ΔB strain generally showed lower individual variation and lower CFU/ml blood compared to the other strains at days 10–13 post infection, indicating some loss of resistance towards host blood immune components.

Taken together these data indicate that in general *As*LPMO10A and -B were not critical for the viability and survival of *A. salmonicida* in salmon blood in the early- or late- stage of infection in vivo*,* albeit *As*LPMO10B was found to be important in the invasive phase of CWV.

### Bacterial burden in tissues and organs

Next, samples were taken from the various tissues and organs to evaluate whether LPMOs were critical for viability of *A. salmonicida* in organs over the course of chronic CWV infection. Assessing the bacterial burden revealed that despite *A. salmonicida* being absent in skin and gills of the sampled fish at day 1–6 post-infection, wild type and ΔA were reisolated from all sampled fish at 8 days post challenge (Fig. 5A, panels 1–2). In the ΔB and ΔAB infected groups, the reisolation was estimated 60–80% and 20–30%, respectively (Fig. 5A, panels 1–2). A quantitative analysis of bacterial burden in the spleen and liver revealed significant increase in the reisolated wild-type compared to the ΔB and ΔAB mutant strains 8 days post-challenge (Fig. 5B, right and left panels). Interestingly, the number of reisolated ΔA strain was attenuated in the spleen (Fig. 5B, right panel), but not liver (Fig. 5B, left panel) at day 8 post-infection. All infected groups showed reduced reisolation of *A. salmonicida* from skin, gills, head kidneys, liver and spleen at the later time-points as the CWV entered into the decline phase. Of note, the ΔB strain was not detected in sampled organs after 8 days post challenge, whereas the ΔAB strain was detected at levels similar to fish challenged with the wild type (Fig. 5A). In summary, these data demonstrate the importance of *As*LPMO10A and -B in the invasive phase of CWV caused by *A. salmonicida.*

### Structure of *As*LPMO10B

**Fig. 6:**
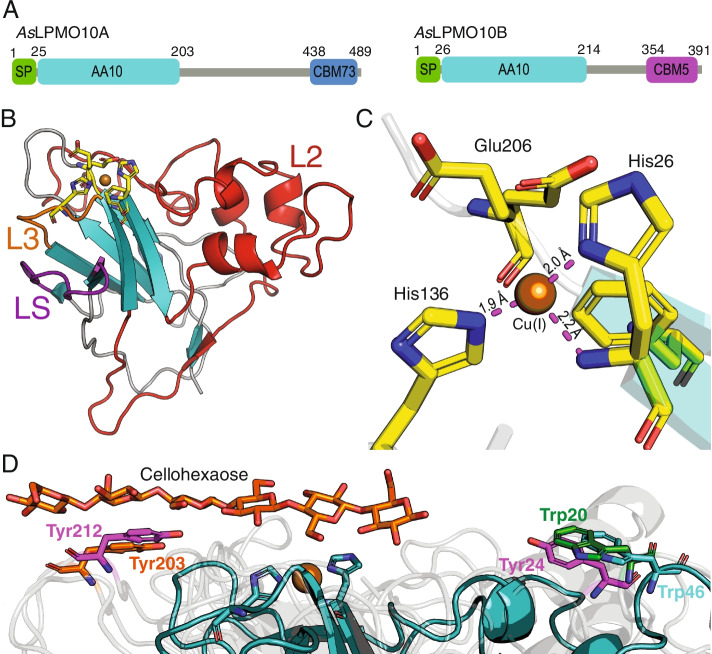
X-ray crystal structure of the *As*LPMO10B AA10 LPMO domain. (**A**) Domain architecture of *As*LPMO10A and -B. Domain boundaries are indicated by amino acid sequence numbers. SP stands for signal peptide, and CBM refers to the carbohydrate binding domain family. (**B**) Cartoon representation of the crystal structure, with the topology assigned as described by Vaaje-Kolstad et al. [47]. The loop short (LS, purple), loop 2 (L2, red), loop 3 (L3, orange) and active site residues (yellow) are indicated by different colors and labeled. (**C**) Active site of *As*LPMO10B, domain 1. The copper ion (bronze) is coordinated by the N-terminus, the side chain Nδ1 of His26 and the side chain Nε1 of His136, forming the so-called histidine brace motif (distance to copper indicated). In the AA10 family, a phenylalanine residue (Phe208) replaces the tyrosine residue found in many other LPMOs, which provides a loose axial coordination for the copper ion. Glu206 was refined in two alternative conformations. In other LPMOs, this residue is often replaced by a glutamine residue (Gln). (**D**) Conservation of aromatic amino acids among LPMOs, as revealed by substrate docking. Loop-2 Trp46 from *As*LPMO10B (teal) is conserved in the viral LPMO fusolin (Trp20; green; PDB ID: 4YN2), a close structural homolog of *As*LPMO10B. Their placement mirrors that of Tyr203 (orange), a substrate-binding residue found on the long C-terminal loop (LC) in *Panus similis* LPMO9A (PDB ID: 5ACI). LPMOs that carry both LC and L2 loops bear aromatic amino acids matching the positions of both Trp46 and Tyr203 (*i.e*. Tyr24 and Tyr212 on *Thermoascus aurantiacus* GH61 isozyme A, colored magenta PDB ID: 2YET). The structures of the three proteins were aligned by secondary structure matching (SSM)

A structural investigation of *As*LPMO10B was initiated to find a rationale for its apparent role as a facilitator during host invasion. The X-ray crystal structure of the family AA10 LPMO domain of the protein (amino acid residues 26–214; Fig. 6A) was solved to a resolution of 1.35 Å (*R*/*R*_free_ = 13.9/16.2%; Table S1) and deposited in the Protein Data Bank (PDB; PDB ID: 7OKR). *As*LPMO10B carries the canonical [47] fibronectin-like/immunoglobulin-like β-sandwich core structure found in other LPMOs (Fig. 6B), consisting of seven β-strands arranged as two juxtaposing β-sheets. The β-sandwich supports the histidine brace catalytic motif (His26, His136) and the putative co-substrate coordinating amino acid (Glu206), which shows conformational heterogeneity and was modeled in two alternative conformations (Fig. 6C). The histidine brace is loaded with a copper ion, as confirmed by anomalous scattering, an expected consequence of the sample preparation process. Copper shows an incomplete square planar coordination, hinting at the presence of Cu (I) at the metal-binding site. The latter is likely a consequence of the well-documented photoreduction of Cu (II) during X-ray data collection [48]. The model also contains 109 water molecules from the first and second coordination sphere and three polyethylene glycol fragments (PEG) from the crystallization conditions. We also observe electron density “above” the copper site (Fig. S1), where the putative ligand would bind, which may represent a citrate molecule from the buffer. A search for structural homologues was run on the DALI server [49] (ekhidna2.biocenter.helsinki.fi/dali), using the coordinates of the new LPMO domain. The list of results contains matches from various members of the LPMO AA10 subfamily, confirming its correct genomic assignment. A visual inspection of the structural alignment with the top ten hits helped to further refine the assignment to the *subcluster 2* described by Vaaje-Kolstad *et A.* [47]. In particular, the distinctive loop 2 (L2) of subcluster 2 is conserved in *As*LPMO10B. This subcluster includes members that display substrate promiscuity for either chitin or cellulose. The match with the highest score (Z score: 27.2, r.m.s.d.: 1.8 Å) was Tma12, a putative AA10 LPMO from the fern *Tectaria macrodonta* (PDB ID: 6IF7; sequence identity: 33%). Tma12 has been proven to shield its host from predators by exerting an entomotoxic activity [50]. Their structural superposition reveals a possible site for *As*LMPO10B *O*-linked glycosylation at Thr166, matching the *N*-linked glycosylation of Tma12 at Asn158. A PEG molecule modeled in close proximity of Thr166 partially superposes with the polar groups of the *N*-linked glycan decorating Tma12, further supporting the hypothesis of *O*-glycosylation.

The AA10 module of *As*LPMO10B also matches several members of subcluster 4, which groups together LPMOs of viral origin. Among them is fusolin from insect poxviruses (PDB ID: 4YN2 [51]) which has 36% sequence identity to *As*LPMO10B and therefore was used as a model for solving the structure (see *Materials and methods*). Their structural alignment (r.m.s.d.: 1.5 Å) shows the conservation of a tryptophan residue on the far edge of L2 (Trp46, Fig. 6D). This tryptophan side chain is oriented parallel to the substrate binding surface and is positioned similar to the tyrosine residue essential for catalysis in the cellulolytic *Panus similis* LPMO9A (Tyr203). In *Ps*LPMO9A, Tyr203 is carried by the long C-terminal loop (LC), absent in both *As*LPMO10B and fusolin, and provides a stacking interaction with the cellulose substrate (Fig. 6D; (PDB ID: 5ACI) [52]). Interestingly, LPMOs that possess both the C-terminal loop and L2, as the *Thermoascus aurantiacus* GH61 isozyme A (PDB ID: 2YET) [53], exhibit aromatic amino acids on both loops, at the position occupied by Trp46 in *As*LPMO10B and Tyr203 in fusolin (Fig. 6D).

## Discussion

To gain insight into the potential roles of chitinolytic enzymes in virulence, the current study set out to elucidate the putative immune evasive properties of *As*LPMO10A and *As*LPMO10B in the pathogenesis of *A. salmonicida* in Atlantic salmon. Given the putative role of LPMOs in mucin binding and attachment of bacteria to mucosal surfaces [32, 54], we hypothesized that *As*LPMO10A and -B could be harnessed in the initial phase of binding to and penetration of the host outer barrier. The fact that the *A. salmonicida* LPMOs are chitin-degrading enzymes [35], combined with the proposed presence of chitin in Atlantic salmon scales [55] makes this hypothesis attractive and highly relevant. A challenge model able to probe all phases of pathogenesis was therefore chosen, namely an immersion challenge where the Atlantic salmon smolts were exposed to *A. salmonicida* in the aqueous environment. Considering that rapid disease development and high mortality may mask potential differences between groups, the selected sub-lethal infection dose was aimed to establish bacteremia without exhaustive killing. Our results indicate that neither of the LPMOs are critical for *A. salmonicida* in passing the outer barrier since all fish were positive for the presence of *A. salmonicida* wild type and deletion variants after 10 min in the challenge bath, and no significant difference between the groups was observed (Fig. 3C). However, the LPMOs were found to be important for the invasive phase of CWV. Particularly *As*LPMO10B showed a significantly lower bacterial burden in blood, spleen and liver compared to the wild type strain 8 days post challenge (Figs. 4 and 5). Similar observations were made for the opportunistic pathogen *Listeria monocytogenes,* where an LPMO deletion strain was attenuated in the spleen and liver three days post systemic infection in mice [25]. The *P. aeruginosa* LPMO (called CbpD) was found to be important for pathogenesis of *P. aeruginosa* over the course of systemic infection via attenuation of the terminal complement pathway [31]. Neither *As*LPMO10A or -B are very similar to CbpD (25.6 and 28.4% sequence identity, respectively), but *As*LPMO10A contains a C-terminal family CBM73 chitin binding domain similar to CbpD (Fig. 6A). Moreover, we note the structural similarity of the *As*LPMO10B AA10 domain with the chitin-active AA10-domain of viral fusolin, an LPMO that strongly enhances the infectivity of entomopoxviruses [51, 56, 57], indicating shared structural features that enable an LPMO to enhance the virulence of a pathogen.

An interesting trait of *A. salmonicida* is its possession of two distinctly different LPMOs. Several other pathogens also share this trait, but many also only carry a single LPMO in their genome (Fig. 1), including *P. aeruginosa* for which the LPMO clearly is a virulence factor [31]. Can it be that the two LPMOs have different functions? Both *A. salmonicida* LPMOs cleave chitin chains by oxidation and contribute to chitin catabolism [35]. On the other hand, *As*LPMO10A is expressed at high abundance in the absence of chitin and has shown a slightly higher rate of chitin oxidation compared to *As*LPMO10B [35]. In the context of the slightly different phenotypes observed for the *As*LPMO10A and-B deletion variants in this study, it is not unlikely that these LPMOs play different roles in *A. salmonicida* pathogenesis.

LPMO deletion variants showed an altered proteome response compared to the wild type, in the presence and absence of Atlantic salmon serum (Fig. 2). Intriguingly, the ΔB and ΔAB strains showed a remarkably higher number of significantly regulated proteins in the presence of the serum compared to the absence of the latter (Fig. 2, panel A). Moreover, general regulation of stress response related proteins, chemotaxis related proteins (ΔA strain), and up-regulation of LuxI in the ΔA and ΔAB strains are intriguing observations (Fig. 2, panel C). The latter protein, LuxI, is important for the regulation of motility and biofilm formation [58]. It should be noted that a substantial proteome alteration was also observed for the *P. aeruginosa* LPMO deletion strain when exposed to human serum (compared to the wild type; [31]), indicating the struggle of the pathogens to interfere with host immune responses when lacking the LPMO(s).

In conclusion, we have shown that the LPMOs of *A. salmonicida* may be moonlighting enzymes that not only contribute to chitin catabolism [35], but also play a role in the pathogenicity of the bacterium in the invasive phase of CWV in Atlantic salmon. Many LPMOs and chitinases of opportunistic pathogens have been shown to depolymerize chitin and also to contribute to chitin catabolism of the bacterium [59, 60]. Therefore, it is likely that chitinolytic enzymes not merely have functions for acquisition of nutrients, but also for protection of the bacteria towards host defense mechanisms.

## Materials and methods

### Bacterial strains

*A. salmonicida* strain LFI1238 originally isolated from the head kidney of an Atlantic cod that died from CWV, and derivative mutant strains (Table 1) were routinely cultivated at 12 °C in liquid Luria Broth (LB) supplemented with 2.5% sodium chloride (LB25; 10 g/L tryptone, 5 g/L yeast extract, 12.5 g/L NaCl) or solid LB25 supplemented with 15 g/L agar powder (LA25). In-frame deletion of *AsLPMO10A*, *AsLPMO10B* and *AsLPMO10A*△*10B* and genes in strain LFI1238 were described in our previous study [35].

**Table 1:** List of bacterial strains

Strain	Description	Ref
LFI1238	*Aliivibrio salmonicida* strain LFI1238	^a^
*As*ΔLPMO10A	LFI1238 containing gene deletion *ΔLPMO10A*	^b^
*As*ΔLPMO10B	LFI1238 containing gene deletion *ΔLPMO10B*	^b^
*As*ΔLPMO10A/Δ10B	LFI1238 containing gene deletions *ΔLPMO10A* and *ΔLPMO10B*	^b^

^a^Originally isolated by the Norwegian Institute of Fisheries and Aquaculture Research, N-9291 Tromsø, Norway, but provided by Simen Foyn Nørstebø for this study

^b^[35]

### Atlantic salmon challenge

All experiments were carried out at the Norwegian Institute for Water Research (NIVA, Solbergstrand, Norway). Fish were monitored daily and upon showing clinical signs of disease during the experimental period were collected and euthanized by an overdose of Benzoak® (ACD Pharmacuticals As, Leknes, Norway).

#### Challenge procedures

The challenge involved 1340 unvaccinated Atlantic salmon parr (average weight 60 g), which were obtained from Center for Fish Research, Department of Animal and Aquacultural Sciences, NMBU. Fish were transported according to the Norwegian Regulations on transport of Aquatic Animals and allocated in their designed experimental groups. Ahead of the immersion challenges, parr-smolt transformation was induced by gradually increasing the salinity of the tank water from 12 to 33 ppm over a period of 11 days, followed by one-week acclimation at 33 ppm. Fish were kept in separate tanks (1400 L) with flow-through of sea water from the Oslofjord (45–50 m depth). The average temperature and salinity of intake water was 9.9 °C and 33.5 ppm respectively. The fish were fed a rate corresponding 1% of the biomass.

The challenge was carried out using 1260 animals randomly divided into 4 experimental groups of 295 fish and one control group (80 fish). The control group was mock challenged with Luria Broth supplemented with 3% NaCl (LB3). The water level was first lowered to 350–400 L. Water intake was temporarily stopped, and ~ 4 L cultures of wild type *A. salmonicida* LFI1238 or LPMO gene deletion strains ΔAsLPMO10A, ΔLPMO10B and ΔAsLPMO10AΔLPMO10B were added directly to the fish tanks. After 30 min the water intake was re-opened and increased to 700 L/h. Water samples were collected before re-opening the water intake. Five to ten live fish from each experimental group were sampled from 10 min into the challenge bath and up to 19 days post challenge. The smolts were monitored for 36 days.

#### Obtaining blood samples from infected fish

For collection of blood samples, fish were anesthetized in a water bath containing benzocaine (Benzoak Vet, ACD Pharmaceuticals AS). Blood was sampled from the caudal vein using the VACUETTE® system and VACUETTE® 4 mL NH Sodium Heparin tubes (Greiner bio-one), 100 and 10 µl of sampled blood was immediately spread onto LA25 in duplicates and incubated at 12 °C 3–5 days**.**

#### Evaluation of bacterial burden in tissues and organs

The bacterial burden was monitored by collection of bacteriological samples 1 h, 4 h, 1 day, 2 days, 4 days, 6 days, 8 days, 10 days, 13 days, 16 days and 19 days post challenge. Samples were collected from skin, gills, spleen, liver and head kidney by using 1 µl sterile disposable inoculation loops (Sarstedt) and patching on LA25 in the following order; midline of skin, outermost lamella of gills, dissection and puncture of spleen, liver and head kidney. Plates were incubated 4–5 days at 12 °C.

#### Persistence of the bacterium in tissue

Tissue intended for RNA isolation was dissected using sterile scalpels and disposable forceps (VWR International), washed twice in Dulbecco’s Phosphate Buffered Saline (PBS, Sigma-Aldritch) and transferred to 15 mL Falcon tubes containing 1–2 mL of protect® Bacteria Reagent (Qiagen). For determination of CFU/mg, the samples were transferred to 2 mL FastPrep® tubes (MP Biomedicals) pre-prepared with 100 µl sterile 1.4 mm ceramic beads (OMNI International) and 200 µl PBS. The tubes were weighed before and after sampling, homogenized by using FastPrep (MP Biomedicals), 4 ms, 3 × 5 s. Volumes of 100 and 10 µl were spread onto LA25 in duplicates and incubated at 12 °C for 3–5 days before calculation of colony forming units/ (mg organ) (CFU/mg).

#### Necroscopy

Euthanized and deceased fish were autopsied to determine the cause of death. External and internal signs were examined, and bacterial samples taken from the head kidney, liver and spleen unless otherwise stated. The bacteriological samples were taken by puncturing the organs with 1 µl sterile disposable inoculation loops (Sarstedt) and streaking onto LA25. *A. salmonicida* was recovered from the head kidney, spleen and liver, in bacteriological samples taken during necroscopy. Culture results were evaluated together with pathological changes such as external and internal hemorrhages, fluid in cavity, discolored liver and swollen spleen.

### Proteomics

Starter cultures of wild type, ∆A, ∆B and ∆AB were grown in LB25, in triplicate, for 48 h at 10 °C with shaking. Next, the cultures were diluted in LB25 to an OD600 of 0.1 and grown until they reached early logarithmic phase (OD_600nm_ = 0.4–0.5). After reaching early logarithmic phase, the cultures were split in two and incubated for 1 h in the absence or presence of 1% Atlantic salmon serum (SS). Thereafter, 1 mM beta-glycerophosphate (Sigma), 1 mM sodium orthovanadate (Sigma), 20 mM sodium pyrophosphate (Sigma), 1 mM phenylmethylsulfonyl fluoride (PMSF, Sigma), and 1 × Complete Mini EDTA-free protease inhibitors (Roche) were added to the samples. The bacterial pellets and supernatants were separated by centrifugation (4500 × *g*, 15 min, 4 °C). The pellets were washed once with PBS and centrifuged, before being resuspended in lysis buffer containing 20 mM Tris–HCl (pH 7.5), 0.1 M NaCl, 1 mM EDTA, 1 × Complete Mini EDTA-free protease inhibitors, and lysozyme (0.5 mg·ml^−1^). Cells were disrupted by sonication (20 × , 5 s off-5 s on, 26% amp), and the cellular debris was cleared by centrifugation (4500 × g, 30 min, 4 °C).

The protein samples were boiled in NuPAGE LDS sample buffer and 30 mM dithiothreitol (DTT) for 5 min before being loaded onto Mini-PROTEAN® TGX Stain- Free™ Gels (Bio-Rad laboratories, Hercules, CA, USA). The gels were run at 200 V for 5–10 min using the BIO-RAD Mini-PROTEAN® Tetra System. The gels were stained with Coomassie brilliant blue R250 and cut into small gel pieces, which were transferred to 2 mL LoBind tubes. The gel pieces were washed in 200 µL of water for 15 min and decoloured by 200 µL 50% acetonitrile (ACN), 25 mM ammonium bicarbonate (AmBic) at room temperature (RT) for 15 min. Decolouring was performed twice. After washing and decolouring, the gel bits were left to shrink and dehydrate for 5 min in 100 µL 100% ACN. In order to reduce and alkylate the proteins, the gel pieces were first incubated for 30 min at 56 °C in a solution containing 10 mM DTT and 100 mM AmBic, and then with 55 mM iodo-acetamide and 100 mM AmBic for 30 min at RT. Thereafter, the gel pieces were dehydrated using 100% ACN and digested overnight at 37 °C in a solution containing 0.3 µg of trypsin. The next day, the digestion was stopped by adding 70 µL 0.5% trifluoroacetic acid (TFA). For peptide extraction, the gel pieces were sonicated for 10 min and afterwards centrifuged (16 000 × *g*, 5 min). The supernatants were then transferred to the StageTips for desalting. This procedure was repeated once more, however for the second round the gel pieces were added 70 µL 0.1% TFA before sonication.

For desalting and cleaning up the extracted peptides, StageTips were used. These were made accordingly: Using an 18 g blunt-ended needle, two pieces of Empore C18 membrane (6683-U, Sigma) were cut out. By a length of 1/32″ peeksil capillary or equivalent, the membrane pieces were pushed firmly into a 200 µl pipette tip. The StageTips were mounted onto LoBind tubes, by a hole in the lids, which were cut out beforehand [61]. The tips were activated by transferring 50 µL of methanol to the tips. Afterwards, the tubes were centrifuged (2500 × *g*, 5 min), and the flowthrough was discarded. For equilibration, 100 µL of 0.1% TFA were added and centrifuged as before. The flowthrough was discarded, and the peptide solution was loaded into the tips after sonication, as described in the previous Sect. 100 µL of 0.1% TFA were added, centrifuged as earlier, and the flowthrough removed. For eluting the peptides, 50 µL of a solution containing 80% ACN and 0.1% TFA were added and centrifuged as above. The peptides were evaporated using a SpeedVac system until dryness. Afterwards, the peptides were redissolved in 12 µL of a solution containing 0.05% TFA and 2% ACN.

The peptides were separated by a nano UPLC (nanoElute, Bruker) operating a C18 reverse-phase column, using a pre-installed program with a 120 min gradient, and analyzed by a trapped ion mobility spectrometry and a quadrupole time of flight mass spectrometer (timsTOF Pro, Bruker). 200 ng of each sample was loaded into the UPLC MS/MS system. The raw files were processed with MaxQuant (version 1.6.17.0) for label-free quantification (LFQ) and searched against the UniProt *A. salmonicida* proteome: UP000001730. The digestion mode was set to specific with Trypsin/P as the digestive enzyme, and a maximum of two missed cleavages were allowed. “Match between runs” was applied using default parameters and the peptides were filtered with a 1% level false discovery rate (FDRs) using a revert decoy database. Carbamidomethylation of cysteines were included in the search as a fixed modification, while protein N-terminal acetylation, oxidation of methionines and deamidation of glutamines were included as variable modifications. For data analysis Perseus version 1.6.15.0 was used, and the quantitate values were log_2_ transformed. Valid values were filtered with minimum 2 values in each group for each of the comparisons, and missing values were imputed. Significantly up- or downregulated proteins were determined by performing Student’s *t*-test (*p* = 0.05). For the volcano plots, differentially expressed proteins were defined by having *p*-values of ≤ 0.05 (log_10_ = 1.3) and log2 fold change > 1.5 (log_2_ = 0.58).

### Protein production and purification

The AA10 domain of *As*LPMO10B was subcloned in the pNIC expression vector by adding a stop codon directly after the codon representing amino acid 217 (D217) in the original *As*LPMO10B expression construct described in [35]. Expression and periplasmic extraction of the protein was performed identically to the protocol described in [35]. The protein was purified using a three-step protocol with chilled buffers and columns or at 4 °C. First, the periplasmic extract was adjusted to the IEX running buffer (20 mM MES pH 5.5, 0.1 mM EDTA) and loaded onto an equilibrated 5 mL HiTrap Q FF column (Cytiva) with a flow rate of 6 mL/min. After unbound protein had passed the column, the bound protein was eluted by applying a linear gradient to 500 mM NaCl in 250 mL. Fractions containing *As*LPMO10B were collected, adjusted to the HIC running buffer (1 M (NH_4_)_2_SO_4_, 20 mM Tris–HCl pH 8.0, 0.1 mM EDTA) and further purified using an equilibrated 5 mL HiTrap Phenyl FF (HS) column. The protein was loaded at 3 mL/min. After unbound protein had passed, the bound protein was eluted by applying a 200 mL linear gradient to 0 M (NH_4_)_2_SO_4_. The fractions containing *As*LPMO10B were collected and concentrated using an Amicon Ultra-15 Centrifugal Filter Unit with a 10 kDa cutoff (Milipore). Finally, 1.5 mL of the concentrated eluate was run through a Superdex 75 120 mL SEC column (Cytiva) using 20 mM Tris–HCl pH 8.0, 150 mM NaCl, 0.1 mM EDTA as running buffer. Pure AsLPMO10B was collected, concentrated and stored at 4 °C until further use.

### Protein crystallization, X-ray structure determination and refinement

The *As*LPMO10B AA10 domain was crystallized by the hanging-drop vapor diffusion method. Before setting up crystallization trials, the protein was saturated with Cu(II) by adding a threefold molar excess of CuSO_4_ after adding 1 mM CaCl_2_ (to saturate EDTA in the buffer). Excess copper was removed with a HiTrap desalting 5 mL column (GE Life Sciences). The buffer was exchanged to 5 mM Tris–HCl pH 8.0 and subsequently concentrated to 30 mg/mL using Vivaspin 20 (10 kDa molecular mass cutoff) centrifugal concentrators (Sartorius Stedim Biotech GmbH). The concentrated protein was stored at 4 °C until use. Crystallization experiments were prepared in a pre-greased 48-well VDX plate (Hampton Research) and mixed on silanized coverslips with the protein solution in a 1:1 volume ratio. Diffraction-quality crystals grew after 30–60 min incubation at 20 °C, from a reservoir solution containing 0.1 M Na-phosphate/citrate pH 4.2 and 40% v/v PEG 300. Crystals were cryoprotected by complementing the crystallization solution with 25% w/v glucose, flash-cooled in liquid nitrogen and stored in a CX-100 Taylor-Wharton dry shipper for synchrotron data collection.

Diffraction data were collected at the MAX-IV synchrotron (Lund, Sweden), on the BioMAX beamline [62] (Dectris EIGER16M hybrid-pixel detector) [63]. Data collection was carried out at 100 K, at a wavelength of 0.9763 Å, covering a total oscillation range of 360° with 0.1° oscillations. Diffraction data were integrated, merged and truncated using the *EDNA* [64] software pipeline. The integration and scaling output was reindexed using *REINDEX*, a component of the *CCP4* crystallography software suite [65]. Crystals belonged to space group *P*6_5_, with unit cell parameters a = 71.1 Å, b = 71.1 Å, c = 100.3 Å and one molecule in the asymmetric unit. Data collection and scaling statistics are reported in Table S1. The structure was solved by molecular replacement using the program *PHASER* [66] from the *CCP4* suite. The structure of *Wiseana spp. entomopoxivirus* fusolin (PDB ID: 4YN2 [51]) served as search model (36% sequence identity) after removing alternative conformations for all residues using the *CCP4* tool *PDBCUR*, and truncating mismatching portions with *SCULPTOR*, another program from the *CCP4* suite [65]. Refinement was carried out using data to 1.35 Å, alternating between cycles of real-space refinement using *Coot* [67] and maximum likelihood refinement against anomalous data with *REFMAC5* [68]. The molecular replacement output was examined and improved by first removing ill-defined side chains and loops, and thereafter adding missing structural elements in a step-wise fashion as the quality of the electron density map improved. After improving the protein main chain, water molecules were added based on compatible electron density and hydrogen-bonding interactions. A peak in the phased anomalous difference map confirmed the presence of copper bound in the center of the histidine brace motif (Fig. S1). Toward the end of the refinement process, missing side chains and alternative conformations were added, and their relative occupancies refined with *PHENIX*.refine [69]. As the last step, the very high data-to-parameter ratio (~ 32) allowed full anisotropic *B*-factor refinement, including ligands and water molecules. The coordinates and structure factors are deposited in the PDB [70] with PDB ID: 7OKR.

### Phylogenetic analysis

Amino acid sequences of LPMO-encoding genes were obtained through the CAZy database [13]. Only protein sequences from known fish pathogens [37] were selected. Before analysis, signal peptides, predicted by SignalP 5.0, were removed from the sequences. The phylogenetic analysis was performed using the “build” function of ETE3 v3.1.1 21 [38] which employs PhyML v20160115 [39], with default parameters. Branch support values were computed from 100 bootstrapped trees.

## Supplementary Information

**Additional file 1:** **Figure S1.** Stereo view of the unmodeled electron density at the catalytic center. The σ_A_-weighted 2m*F*o-*F*c electron density around the histidine brace is contoured at 1.0 σ; the phased anomalous difference density map contoured at 4.0 σ shows a large peak, modeled as a copper ion. The figure includes a large peak in the σ_A_-weighted *F*o-*F*c map contoured at 3.0 σ, visible between the histidine brace and the putative catalytic base (Glu206) and left unmodeled. **Table S1.** Data collection and refinement statistics

**Additional file 2:** 

**Additional file 3:** 

**Additional file 4:** 

## Data Availability

The proteomics data obtained in this study is available in the PRIDE database (https://www.ebi.ac.uk/pride/) with the accession number PXD031437. The X-ray crystallographic structure and accompanying data of the *As*LPMO10B AA10 domain has been deposited in the pdb database (www.rcsb.org) with the PDB ID: 7OKR.
