# Supplementary material for: Chitinolytic enzymes contribute to the pathogenicity of Aliivibrio salmonicida LFI1238 in the invasive phase of cold-water vibriosis
Source: BMC Microbiol. 2022 Aug 8;22:194. doi: 10.1186/s12866-022-02590-2 (PMC9361615; doi:10.1186/s12866-022-02590-2)
Supplement: Supplementary file 1 — Additional file 1: Figure S1. Stereo view of the unmodeled electron density at the catalytic center. The σA-weighted 2mFo-Fc electron density around the histidine brace is contoured at 1.0 σ; the phased anomalous difference density map contoured at 4.0 σ shows a large peak, modeled as a copper ion. The figure includes a large peak in the σA-weighted Fo-Fc map contoured at 3.0 σ, visible between the histidine brace and the putative catalytic base (Glu206) and left unmodeled. Table S1. Data collection and refinement statistics [file 12866_2022_2590_MOESM1_ESM.docx]

**Chitinolytic enzymes contribute to the pathogenicity of *Aliivibrio salmonicida* LFI1238 in the invasive phase of cold-water vibriosis**

Anna Skåne^1^, Per Kristian Edvardsen^1^, Gabriele Cordara^3^, Jennifer S.M. Loose^1^, Kira D. Leitl^3^, Ute Krengel^3^, Henning Sørum^2^, Fatemeh Askarian^4 ‡*^and Gustav Vaaje-Kolstad^1‡*^

^1^Faculty of Chemistry, Biotechnology and Food Science, Norwegian University of Life Sciences (NMBU), Ås, Norway.

^2^Department of Paraclinical Sciences, Faculty of Veterinary Medicine, Norwegian University of Life Sciences (NMBU), Oslo, Norway.

^3^Department of Chemistry, University of Oslo, P.O. Box 1033 Blindern, NO-0315 Oslo, Norway.

^4^Division of Host-Microbe Systems & Therapeutics, Department of Pediatrics, School of Medicine, UC San Diego, La Jolla, CA, USA.

^‡^These authors contributed equally to this work.

^*^Correspondence: [gustav.vaaje-kolstad@nmbu.no](mailto:gustav.vaaje-kolstad@nmbu.no), [fatemeh.askarian@nmbu.no](mailto:fatemeh.askarian@nmbu.no)

**Supplementary data**


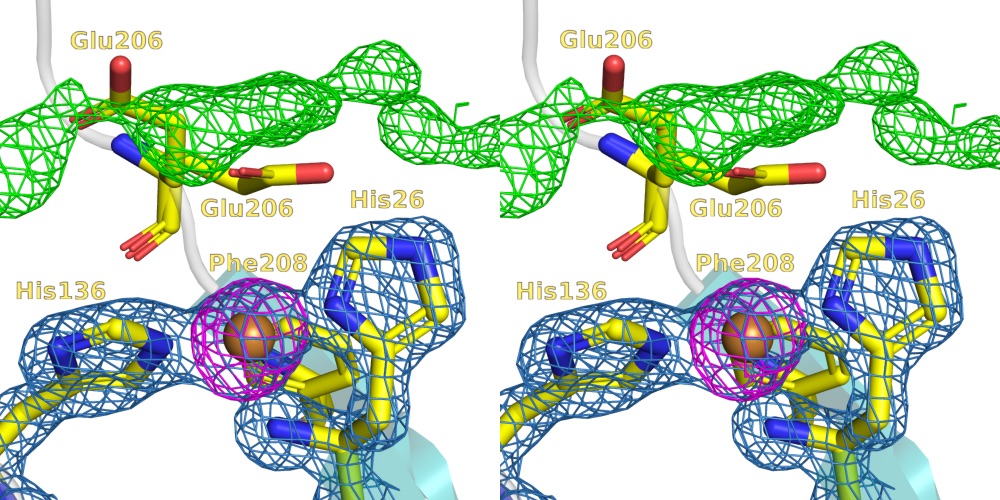


**Figure S1. Stereo view of the unmodeled electron density at the catalytic center.** The σ_A_-weighted 2m*F*o-*F*c electron density around the histidine brace is contoured at 1.0 σ; the phased anomalous difference density map contoured at 4.0 σ shows a large peak, modeled as a copper ion. The figure includes a large peak in the σ_A_-weighted *F*o-*F*c map contoured at 3.0 σ, visible between the histidine brace and the putative catalytic base (Glu206) and left unmodeled.

|  |  |
| --- | --- |
|  |  |
| **Table S1 - Data collection and refinement statistics** | |
|  |  |
|  |  |
|  | *As*LPMO10B-D1^a^ |
|  |  |
|  |  |
| **A. Data collection** |  |
| Beamline | MAX-IV BioMAX |
| Wavelength (Å) | 0.9763 |
| Space group | *P*6_5_ |
| Cell parameters – *a*, *b*, *c* (Å) | 71.1, 71.1, 100.3 |
| Resolution (Å)^b^ | 33.5-1.35 (1.40-1.35) |
| *R*_merge_ (all *I*^+^ and *I*^-^) (%)^bc^ | 9.7 (>100) |
| *R*_merge_ (within *I*^+^/*I*^-^) (%)^bc^ | 9.3 (>100) |
| *R*_meas_ (all *I*^+^ and *I*^-^) (%)^bd^ | 9.9 (>100) |
| *R*_meas_ (within *I*^+^/*I*^-^) (%)^bd^ | 9.8 (>100) |
| *R*_p.i.m._ (all *I*^+^ and *I*^-^) (%)^be^ | 2.2 (54.5) |
| *R*_p.i.m._ (within *I*^+^/*I*^-^) (%)^be^ | 3.1 (83.0) |
| CC_1/2_^bf^ | 99.9 (54.5) |
| Mean I / σ(I)^b^ | 15.3 (0.9) |
| Completeness (%)^b^ | 99.9 (99.7) |
| Multiplicity^b^ | 18.2 (9.7) |
| No. reflections (unique) | 62730 (6133) |
| Wilson *B*-factor (Å^2^) | 20.2 |
|  |  |
| **B. Refinement** |  |
| Resolution (Å) | 33.5-1.35 |
| *R*_work_/*R*_free_ (%)^g^ | 13.9 / 16.2 |
| Macromolecules / a.s.u. | 1 |
| *No. atoms* |  |
| Protein | 1715 |
| Water | 109 |
| Ligands | 22 |
| *B-*factors *(Å^2^)* |  |
| Protein | 28.5 |
| Water | 37.3 |
| Ligands | 66.3 |
| *r.m.s.d. from ideal values* |  |
| Bond lengths (Å) | 0.02 |
| Bond angles (deg.) | 1.86 |
| *Ramachandran plot* |  |
| Core region (%) | 97.9 |
| Outliers (%) | 0 |
| PDB ID | 7OKR |
|  |  |
|  |  |

^a^Friedel pairs were treated as different reflections.

^b^Values in parentheses refer to highest resolution shell

^c^*R*_merge_ = Σ**_h_**Σ*_j_* |*I***_h_***_j_* - 〈*I***_h_**〉| / Σ**_h_**Σ*_j_* *I***_h_***_j_* , where 〈*I***_h_**〉 is the mean intensity of symmetry-related reflections *I***_h_**

^d^*R*_meas_ = Σ**_h_** [N**_h_**/(N**_h_**-1)]^1/2^ Σ*_i_* |*I***_h_***_j_* - 〈*I***_h_**〉| / Σ**_h_**Σ*_i_* *I***_h_***_j_* , where N is the redundancy of reflection **h**

^e^*R*_p.i.m._ = Σ**_h_** [1/(N**_h_**-1)]^1/2^ Σ*_j_* |*I***_h_***_j_* - 〈*I***_h_**〉| / Σ**_h_**Σ*_j_*  *I***_h_***_j_*

^f^The high resolution cut-off was chosen by visual inspection of the electron density map, ensuring the presence of a low signal-to-noise ratio

^g^*R*_free_ was calculated from 5% of randomly selected reflections for each data set
